# *Amfor*-Mediated cGMP-PKG Signaling and Transcriptomic Divergence Underlying Division of Labor in *Apis mellifera ligustica*

**DOI:** 10.3390/insects17060583

**Published:** 2026-06-03

**Authors:** Zongwen Hu, Daohao Xie, Xu Dai, Juan Yang, Chunhui Miao, Fangdong You, Jun Liu, Yahui Li

**Affiliations:** 1 Institute of Sericulture and Apiculture, Yunnan Academy of Agricultural Sciences, Caoba 661101, China; 2 Faculty of Animal Science and Technology, Yunnan Agricultural University, Kunming 650201, China; 3 Livestock Chief Station of Yunnan Province, Kunming 650224, China; 4 Puer Milin Biotechnology Co., Ltd., Puer 665000, China

**Keywords:** western honeybee, division of labor, *foraging*, cGMP-dependent protein kinase, proboscis extension response, foraging task, defense

## Abstract

Traits such as defense and foraging in social insects depend on the coordinated division of labor (DOL) among workers. However, several aspects of the molecular mechanisms driving behavioral specialization for these tasks remain incompletely characterized. In this study, we examined two forms of DOL in the Western honeybee (*Apis mellifera ligustica*): foraging (nectar, pollen, and water collection) and defense (guard bees). Using proboscis extension response (PER) assays, gustatory response score (GRS), quantitative PCR, enzyme-linked immunosorbent assay, and transcriptome RNA sequencing of brain tissue, we characterized the behavioral and molecular differences among four task groups. Water foragers showed the highest PER values, gustatory response scores, *Amfor* expression, and PKG activity, while guard bees showed the lowest PKG activity. Transcriptome analysis identified up to 418 differentially expressed genes (DEGs) between forager subtypes and guard bees. DEGs in water foragers were associated with body surface morphology and water transport, those in pollen and nectar foragers with cGMP synthesis, and those in guard bees with retinol metabolism and olfaction. KEGG enrichment analysis of DEGs from guard-vs-forager pairwise comparisons identified the cGMP-PKG signaling pathway as significantly enriched in both foraging-associated and defense-associated DEG lists, indicating that this pathway serves as a shared outside-hive behavioral enabler rather than a foraging-specific switch. WGCNA further revealed that *Nos*, *Camkii*, and *Amfor*-encoded PKG show correlated expression patterns within the same co-expression module, suggesting that a broader calcium-NO-cGMP signaling network, rather than *Amfor* alone, may constitute the functional molecular unit underlying task-specific behavior. These findings provide a transcriptomic framework for understanding how the cGMP-PKG pathway and its associated network regulate behavioral DOL in social insects.

## Simple Summary

Social insects such as honeybees rely on coordinated division of labor (DOL) between workers for colony survival, with foraging and nest defense being two core tasks. However, the molecular mechanisms driving behavioral specialization for these tasks remain poorly understood. Here, we investigated foragers (nectar, pollen, and water collectors) and guard bees of the Western honeybee (*Apis mellifera ligustica*) using behavioral assays and transcriptomic approaches. We found that the proboscis extension response (PER) and gustatory response scores (GRS) were significantly higher in foragers (especially water foragers) than in guard bees. *foraging* (*for*) gene expression levels and PKG activity were also higher in foragers than in guard bees, with pollen foragers showing higher levels than nectar foragers. Transcriptomic analysis further identified distinct task-related gene expression patterns and highlighted the cGMP-PKG signaling pathway as a key regulator of DOL. Our findings reveal that *Amfor* and PKG signaling suggest a role in task specialization in honeybees, providing new insights into the conserved regulation of social insect behaviors.

## 1. Introduction

Division of labor (DOL) is a key characteristic of social insect colonies among sterile worker insects, which form the vast majority of colony members. DOL enables colonies to exploit environmental resources with remarkable efficiency [1,2]. Considerable progress has been made in characterizing the molecular [3], physiological [4], neurological [5], and developmental [6] mechanisms regulating DOL across social species; however, how these mechanisms interact to produce task-specific behavioral specialization in different ecological and social contexts remains incompletely understood.

The honeybee colony, a well-characterized model of social DOL, has developed an efficient group survival strategy through specific tasks such as nutritional foraging (nectar and pollen collection), non-nutritional foraging (water and propolis collection), and defense (guard bees) [7,8]. These specialized individuals respond to various gustatory stimuli at the behavioral level. Nectar foragers adjust their selectivity for nectar sources based on the colony’s nutritional status [9,10]. Pollen collection is facilitated by hierarchical recruitment systems, in which negative feedback from pollen stores within the colony suppresses further collection when reserves are sufficient [11]. Water foragers regulate collection frequency according to colony needs [12]. Guard bees recognize non-nestmate chemical signals at the hive entrance, which trigger aggressive defensive behavior to protect the colony [13,14]. Overall, changes in resource perception during foraging and defense contribute to task specialization in honeybees [15]. These behaviors are essential for their environmental adaptation and serve as a core entry point for understanding complex behavioral regulatory mechanisms in social insects.

Interestingly, honeybee DOL is a well-characterized model system, typically following temporal polyethism in which young worker bees initially perform in-hive tasks (e.g., brood care, nest cleaning), while older individuals transition to outside-hive tasks, including foraging (nectar, pollen, water) and defense [4]. Foragers are usually older than guard bees [16,17]. From a molecular perspective, the *foraging* gene (*Amfor*), which encodes a cGMP-dependent protein kinase (PKG), has been shown to influence key decisions related to insect behavior [18,19]. In social bees, *Amfor* contributes to the transition from internal tasks to foraging outside the hive [20,21]. It also affects levels of dance activity [22], gustation, phototaxis, and locomotion [23]. However, behavioral outcomes often arise from interactions among several genes rather than from a single gene [24]. Transcriptomic and candidate gene studies have identified multiple regulators of DOL, including acetylcholinesterase (*Ache*) [25], the *foraging* gene [26], and the manganese transporter *malvolio* (*mal*) [27]. As for the *Amfor* gene, it is not clear whether it may act as a crucial trigger for the honeybee outside-hive tasks transition from guarding to foraging.

Despite this progress, two important questions remain unresolved. First, prior studies have focused predominantly on the nurse—forager transition, leaving the molecular basis of the guard bee—forager transition largely uncharacterized. Guard bees perform an outside-hive defensive task but are developmentally younger than foragers, raising the question of whether *Amfor* expression follows the same trajectory in defensive as in foraging contexts [3]. In ants, PKG activity is higher in defensive morphological castes (majors) than in foraging minors, suggesting that the relationship between *for* and defense may differ across species and task types [28]. Whether a similar divergence exists between guard bees and forager subtypes in *A. mellifera* has not been directly tested. Second, the transcriptomic landscape that distinguishes not only foragers from guards, but also the three forager subtypes (nectar, pollen, water) from one another, has not been characterized at the whole-transcriptome level in a single integrated study. We hypothesize that *Amfor*-mediated cGMP-PKG signaling differentiates guard bees from forager subtypes at both the molecular and transcriptomic levels underlying task specialization in honeybee division of labor.

The central question of this study is whether *Apis mellifera ligustica* workers performing four naturally occurring tasks—nectar foraging, pollen foraging, water foraging, and colony defense—differ in their gustatory responsiveness, *Amfor* expression, PKG activity, and whole-brain transcriptomic profiles, and if so, what molecular and pathway-level signatures characterize each task group. To address this, we assessed gustatory responses using PER and GRS assays, quantified *Amfor* expression and PKG activity, and performed whole-brain RNA-seq with downstream enrichment and co-expression network analyses. Our goal was to define the molecular and transcriptomic basis of task specialization across both foraging subtypes and defense, and to evaluate the role of *Amfor*-mediated cGMP-PKG signaling within this broader context.

## 2. Materials and Methods

### 2.1. Test Materials and Experimental Sites

**Figure 1:**
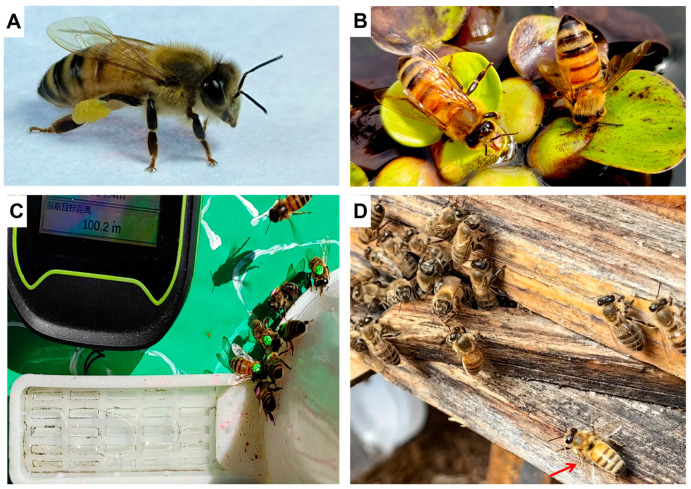
Adult worker bees performing the behavioral tasks analyzed in this study. (**A**) Pollen forager of *A. m. ligustica* (mP); (**B**) Water forager of *A. m. ligustica* (mW); (**C**) Nectar forager of *A. m. ligustica* (mN), “当前目标距离” refers to the distance from the beehive to the feeder; (**D**) Guard bee of *A. m. ligustica* (mG). The red arrow in panel (**D**) indicates a guard bee displaying the characteristic defensive posture (lowered head, raised abdomen, and open wings) in response to the introduced wasp stimulus.

This study was conducted at the apiaries of the Eastern Bee Research Institute of Yunnan Agricultural University (25.129079 °N, 102.751322 °E, Kunming, China) and the Sericulture and Apiculture Institute of Yunnan Academy of Agricultural Sciences (23.525908 °N, 103.40023 °E, Caoba, China) from March 2023 to November 2024. Three *Apis mellifera ligustica* (abbreviated as *A.m.ligustica*) colonies were selected. Each colony served as an independent biological replicate. Bees from each colony were collected and processed separately at all stages, including RNA extraction, library preparation, and qPCR. The three libraries per task group (e.g., mN_1, mN_2, mN_3) correspond to colonies 1, 2, and 3, respectively. Each colony was led by a six-month-old queen, contained approximately 50,000 workers, and had adequate honey and pollen reserves to sustain the bees’ normal activities. The colonies were kept in a Langstroth hive and managed following the standard beekeeping practices. This study focused on DOL among adult worker bees, specifically examining their roles in nectar, pollen, and water foraging and guarding activities (Figure 1). Western honeybees forage propolis, yet individuals that perform this task are difficult to capture under the ecological conditions at that time. Thus, propolis foragers were not studied in this research.

To collect worker bees performing specific tasks, we identified individuals based on their foraging and guarding behaviors. Numerous bees returned to the hive with pollen early in the morning, with colorful pollen clearly visible on pollen baskets at the hive entrance. We temporarily closed the hive entrance to allow several pollen collectors to gather and then captured bees carrying substantial pollen loads, identifying them as pollen foragers. Water and nectar foragers are indistinguishable at the hive entrance upon their return. To confirm that collected nectar foragers originated from the focal colonies, bees were individually stained as many as possible with POSCA Marker (Mitsubishi Pencil Co., Ltd., Tokyo, Japan) before experiment in the hive. Only marked individuals were collected at the feeder and water tank during the testing period. To identify nectar foragers, we placed a feeder containing a 2 M sucrose solution 100 m away from the hive to train bees to visit. Once we observed over 50 bees at the feeder, we captured those that arrived and classified them as nectar foragers. For water foragers, we installed a water tank near the apiary. During hot midday hours, numerous bees were observed foraging for water around the tank, and we identified and captured these bees as water foragers. To identify guard bees, we caught a wasp, attached a wire around its waist to allow free movement, and approached the hive entrance with it. *A. mellifera* displayed defensive behaviors such as lowering heads, raising hips, opening wings, and exposing the Nasonov gland. We captured such bees exhibiting defensive behaviors, classifying them as guards [29].

### 2.2. Bee Capture and Fixation

We captured the bees using a tube, either at the hive entrance, at the edge of the water tank, or at the feeder. The captured bees were transported to the laboratory and immediately placed on ice for 3 min and 30 s to immobilize them. Bees were individually restrained in test tubes, allowing their heads to move freely. Before the proboscis extension response (PER) test, the bees were fed with 30% (*w*/*w*) sucrose solution until satiation and then maintained at 75% relative humidity (RH) and 25 °C for 90 min to exclude bees in poor condition. For the qPCR test, the bees were collected in a frozen storage tube, immediately placed in liquid nitrogen, and then stored at −80 °C.

### 2.3. PER Test

Following the method described by Scheiner et al. [30], we assessed the PER by stimulating the honeybee antennae for 3 s with sugar solutions at seven sucrose concentrations (0%, 0.1%, 0.3%, 1%, 3%, 10%, 30%). To avoid intrinsic sensitisation, we maintained a 2 min interval after each stimulation before applying the next sucrose concentration. This process was repeated three times independently for each task group, with 24 bees used per biological replicate.

### 2.4. Analysis of Amfor Gene Expression Among Task Groups by RT-qPCR

Honeybees were removed from the −80 °C freezer and transferred to the experimental workbench in liquid nitrogen. After the brains of 12 honeybees per task group per replicate were removed under ice-cold sterile conditions. Total RNA of tissues were extracted using Trizol (Shengong, Shanghai, China) following the manufacturer’s protocol. To eliminate potential genomic DNA contamination, extracted RNA was treated with RNase-free DNase I (Thermo Fisher Scientific, Waltham, MA, USA) for 30 min at 37 °C prior to cDNA synthesis. RNA integrity was confirmed by 1% agarose gel electrophoresis and NanoDrop^TM^ 2000 spectrophotometry (Thermo Fisher Scientific). cDNA was synthesized using the HiScript III RT SuperMix for qPCR (+gDNA wiper) kit (Yeasen Biotechnology Co., Ltd., Shanghai, China) following the manufacturer’s instructions. The nucleic acid was diluted with nuclease-free water to 1 μg/μL in a volume of 10 μL. qPCR was used to determine the relative expression level of the target gene *Amfor* related to DOL by using an Applied Biosystems StepOnePlus Real-Time PCR System (Applied Biosystems, Foster City, CA, USA). The primers for the *Amfor* gene (NCBI Gene ID: 406092) were: *Amfor*-F: TTGCCTGGTGATAGACCGTG and *Amfor*-R: TCGCTCTATTCTCCCATCCTTC. The reference gene were *Amef1α* (NCBI Gene ID: 544670) [31]: *Amef1α*-F: CGGTATTTTGAAACCAGGTATGCT and *AmEf1α*-R: CACGTAACCACGCCTCAACT. The qPCR reaction system consisted of ChamQ Universal SYBR qPCR Master Mix (Yeasen Biotechnology Co., Ltd., Shanghai, China) (10 µL), forward primer (0.4 µL), reverse primer (0.4 µL), cDNA template (2 µL), and ddH_2_O (7.2 µL). The reaction process was as follows: pre-denaturation at 95 °C for 5 min; denaturation at 95 °C for 2 min; 40 cycles of (95 °C for 10 s, annealing temperature (Tm) for 30 s, 72 °C for 45 s); extension at 72 °C for 10 min.

Primers were designed using Primer Premier 5.0 software. 2^−ΔΔCT^ method was used to analyze data. Statistical differences in relative gene expression among task groups were assessed by one-way ANOVA followed by Tukey’s HSD post hoc test (*p* < 0.05) using SPSS 19.0 (IBM Corp., Armonk, NY, USA).

### 2.5. Determination of PKG Enzyme Activity in Honeybee

For each task group, brains from ten honeybees per colony were independently homogenized using a pestle homogenization in 650 µL of normal saline on ice, yielding three independent biological replicates per task group (one per colony); samples were then centrifuged at 3000× *g* for 10 min at 4 °C to collect the supernatant. The insect cGMP-dependent protein kinase (PKG) ELISA detection kit (Jingmei Biotechnology Co., Ltd., Yancheng, China) was used to measure PKG enzyme activity in honeybee brains according to the instruction manual. Each task group was tested three times.

### 2.6. RNA Extraction, cDNA Library Construction, and mRNA Sequencing

Sequencing was performed on pooled samples of 12 bee brains per task, using 3 independent colony-derived biological replicates. Following the RNA extraction procedure described in Section 2.4, RNA integrity was examined by agarose gel electrophoresis, and the RIN value was determined using Agilent 2100 (Agilent Technologies Inc., Santa Clara, CA, USA). RNA with a poly(A) tail was then reverse transcribed (first-strand synthesis) using Oligo(dT) primer and reverse transcriptase (Moloney murine leukemia virus). The second strand of cDNA was synthesized using a TSO (template-switching oligo) primer, displacing RNA complementary to the first-strand cDNA. The cDNA was purified using the QIAquick PCR Purification Kit (QIAGEN China (Shanghai) Co., Ltd., Shanghai, China), eluted with elution buffer, and subjected to end repair and poly(A) tail addition. Modified highly active Tn5 transposase was used to fragment DNA while adding adapters to both ends of the cDNA. Products with adapters were purified, size-selected, and amplified by PCR. The final library was obtained after purification. The cDNA library was loaded onto the Illumina sequencing platform (NovaSeq × Plus) for sequencing. Libraries were sequenced on the Illumina NovaSeq × Plus platform (Majorbio using paired-end 150 bp (PE150) chemistry).

### 2.7. RNA-Seq Data Quality Control, Preprocessing and Quantification

Raw reads were assessed for quality using FastQC (v0.11.5). Adapter trimming and quality filtering were performed using Fastp (v0.23.2) with the following parameters: adapter auto-detection enabled; reads with >10% ambiguous bases (N ratio > 10%), reads shorter than 20 bp after trimming, and bases with Phred quality score < 20 at the 3′ end were discarded. Filtered reads were aligned to the *A. mellifera* reference genome (Amel_HAv3.1; GCA_003254395.2) [32] using HISAT2 (v2.2.1) with default parameters and the -dta flag to enable downstream transcript assembly. Read counts per gene were obtained using htseq-count (v0.12.3) in Python (v3.5) with the following settings: stranded = reverse, mode = union, minaqual = 10, using the Amel_HAv3.1 GTF annotation file.

### 2.8. Screening of Differentially Expressed Genes (DEGs)

The DESeq2 package (v1.28.1) in R (v4.0.2) was employed to normalize raw counts and obtain gene expression levels [33,34]. Principal Component Analysis (PCA) was performed using all detected genes from the transcriptomic dataset. PCA in R (v4.0.2) was utilized to evaluate overall similarity among samples. DEGs were identified using the DESeq2 package via the Wald test, with the |log_2_FC| ≥ 1 threshold incorporated directly into the composite null hypothesis using the lfcThreshold = 1 argument in the results() function; raw *p*-values were adjusted using the Benjamini–Hochberg (BH) procedure, and genes with FDR-adjusted *p*-value ≤ 0.05 under this test were defined as DEGs. GO and KEGG enrichment analyses were performed using the clusterProfiler package (v3.16.1), with GO annotations retrieved from org. Am.eg.db (v3.14.0) and KEGG pathway associations obtained via the KEGG REST API (ame organism code).

WGCNA was performed using the WGCNA R package (v1.71). Filtered expression data was performed using soft-thresholding power β = 9 (scale-free R^2^ ≥ 0.85), dynamic tree-cutting (minimum module size = 30, minimum kME = 0.3, merge cut height = 0.25), and Pearson correlation for module–trait associations. GSEA was performed using clusterProfiler (v3.16.1), with genes ranked by signed DESeq2 Wald test statistics, gene sets of 15–500 genes, and a threshold of |NES| ≥ 2.0 and adjusted *p* < 0.05 for reported results.

### 2.9. RNA-Seq Validation by RT-qPCR

The remaining RNA samples from transcriptome sequencing were reverse transcribed into cDNA for technical validation of the RNA-seq results. To provide independent biological validation, qPCR was performed on brain RNA extracted from six additional colonies not used in the RNA-seq analysis, using two validated reference genes (*AmEf1α*, Gene ID: 544670, and *Amarf1*, Gene ID: 409481; [35]) for normalization and three target genes (*Amfor*, *AmOct*, Gene ID: 406068, and *AmInR*, Gene ID: 725827). Primer sequences are provided in Supplementary Table S1. Analytical methods are described in Section 2.4.

### 2.10. Statistical Analysis

The data were analyzed and processed using R 4.3.2. The PER was treated as a binary variable (present or absent, 1 or 0) during sensory assays, and the median values (circles) and quartiles (upper and lower lines) of GRS are shown in the graphs. The Kolmogorov–Smirnov test was used to evaluate whether the binary data conformed to a normal distribution. The results indicated that GRS data from labor tasks did not conform to a normal distribution (KS distance _pollen_ = 0.3457, KS distance _nectar_ = 0.2779, KS distance _water_ = 0.4075, KS distance _guard_ = 0.2499, *p* < 0.01). Subsequently, non-parametric ANOVA was used to test the GRS. For GRS datasets with more than three groups, Dunn’s multiple comparisons test was used, and the Kruskal–Wallis test was applied for datasets with two groups [36,37].

For the percent of PER (present/(present + absent) × 100%) and relative mRNA expression levels and enzyme activities, two-way ANOVA was employed. Tukey’s HSD test was used for post hoc comparisons. All data are expressed as mean ± standard deviation (mean ± SD), and *p* < 0.05 was considered statistically significant.

## 3. Results

### 3.1. Behavioral Differences Among Honeybee Tasks

**Figure 2:**
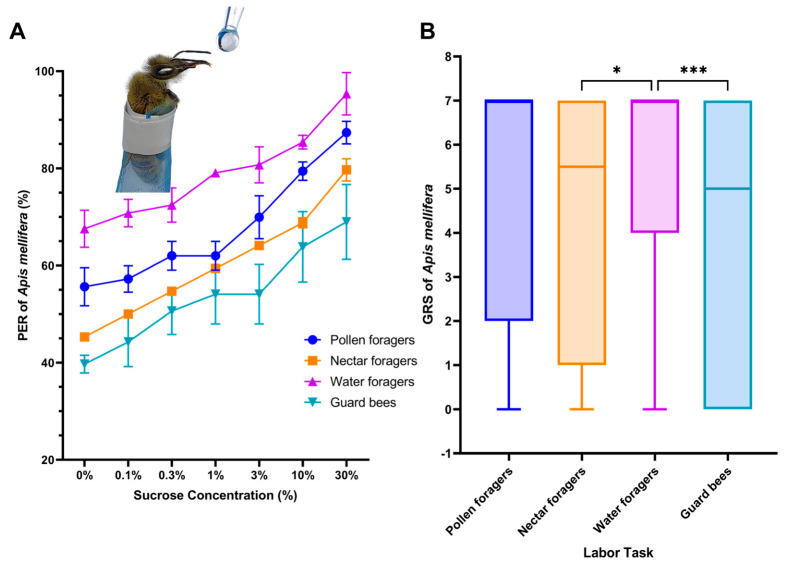
The proboscis extension response (PER, **A**) and gustatory response scores (GRS, **B**) of *A. m. ligustica* under 3 × 24 bees per task group. The results showed that water foragers had significantly higher PER and GRS. Data are presented as mean ± standard deviation (SD) (**A**), and median with 2.5–97.5 percentile whiskers (**B**). * *p* < 0.05; *** *p* < 0.01.

The proboscis extension response (PER) differed between worker bees performing different tasks (Figure 2A, F(3, 24) = 6.811, *p* = 0.0018). The PER of water foragers (78.76 ± 3.625%) was significantly higher than that of nectar foragers (60.28 ± 4.434%, *p* = 0.0202) and guard bees (53.65 ± 3.878%, *p* = 0.0013). There were no significant differences between pollen foragers (67.66 ± 4.495%), nectar foragers, and guard bees (*p* > 0.05, Tukey’s multiple comparison test).

There were also differences in gustatory response scores (GRSs) across tasks (Figure 2B, Kruskal–Wallis statistic = 16.79, *p* = 0.0008). The GRSs of water foragers were significantly higher than those of nectar foragers (*p* = 0.0470) and guard bees (*p* = 0.0005, Dunn’s multiple comparison test).

### 3.2. Variation in Amfor Relative Expression and PKG Enzyme Activity

**Figure 3:**
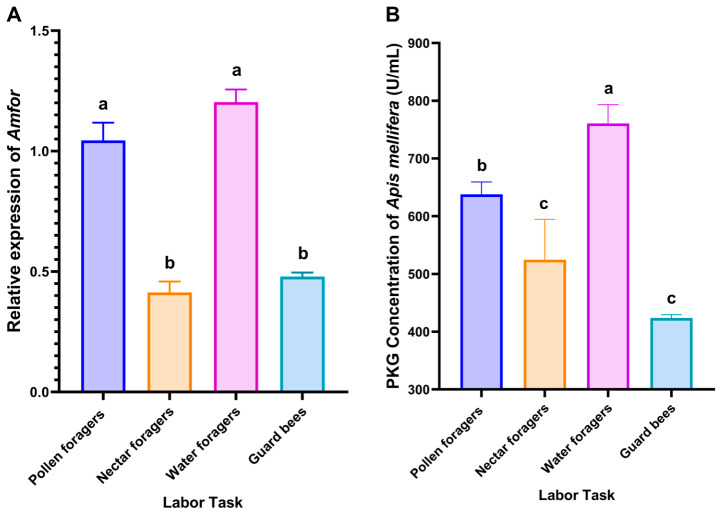
The relative expression level of the *Amfor* gene (**A**) and the concentration of PKG enzyme (**B**) in honeybees among tasks. The data in the figure are presented as the mean ± SD. Different lowercase letters (a, b, c) above bars indicate statistically significant differences between groups (Tukey’s HSD, *p* < 0.05); groups sharing the same letter are not significantly different.

The relative expression levels of the *Amfor* gene in *A. mellifera* varied among tasks (Figure 3A, F (3, 68) = 7.096, *p* = 0.0003). The *Amfor* expression in water foragers was significantly higher than in nectar foragers (*p* < 0.01) and guard bees (*p* < 0.001). Similarly, pollen foragers showed significantly higher expression than nectar foragers (*p* < 0.01) and guard bees (*p* < 0.01). There were no significant differences between water and pollen foragers (*p* = 0.1339) or between nectar foragers and guard bees (*p* = 0.7997).

Furthermore, PKG, encoded by *Amfor*, also differed in *A. m. ligustica* (Figure 3B, F (3, 8) = 39.45, *p* < 0.0001). PKG activity in water foragers (760.8 ± 18.87 U/mL) was significantly higher than in pollen foragers (637.9 ± 12.35 U/mL, *p* = 0.0231). Pollen foragers showed higher PKG activity than nectar foragers (524.9 ± 40.24 U/mL, *p* = 0.0351) and guard bees (423.8 ± 3.227 U/mL, *p* = 0.0008). There was no significant difference between nectar foragers and guard bees (*p* = 0.0585). These results indicated that the foraging-related gene *Amfor* played a central role in regulating honeybee behaviors associated with resource collection (water, pollen, nectar) and defense.

### 3.3. RNA Sequencing Quality Control

A total of 12 cDNA libraries were obtained from three biological replicates in each group: nectar foragers, pollen foragers, water foragers, and guard bees (Table 1). The number of filtered reads in these 12 samples ranged from 45,327,254 to 59,340,992. A total of 97.79 GB of clean data was obtained through transcriptome analysis, with each sample yielding more than 6.77 GB. The GC content of all samples was between 36.92% and 39.14%. Therefore, the sequencing data were suitable for subsequent analysis. The unique mapping ratio ranged from 86.01% to 93.89%.

### 3.4. Sample Correlation and PCA

**Figure 4:**
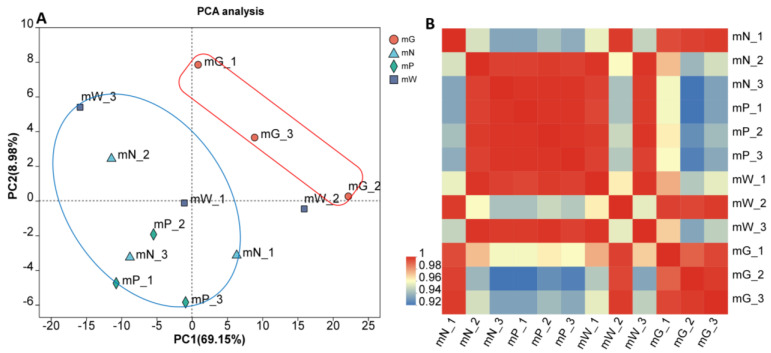
Principal component analysis (**A**) and sample correlation analysis (**B**). mN, nectar forager of Western honeybees, *A. m. ligustica*; mP, Pollen forager; mW, water forager; mG, Guard. Rectangles and ellipses (**A**) are drawn manually for visual grouping purposes only and do not represent statistically derived confidence regions.

Based on the whole expression matrix, PCA and correlation analysis between samples were conducted. In the PCA score plot, PC1 and PC2 explained 78.13% of the total variation, showing two distinct clusters representing foraging and defense tasks in Western honeybees. However, replicate group 2 in the water forager group exhibited noticeable separation (Figure 4A). In the sample correlation analysis, squares of different colors represent correlation levels between samples, indicating a high correlation among biological replicates (Figure 4B).

### 3.5. DEGs Across Labor Tasks

**Figure 5:**
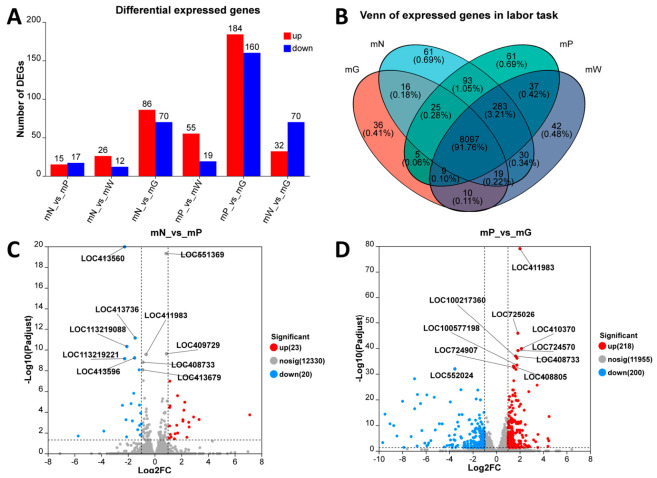
DEG analysis. (**A**) Venn of expressed genes in four labor tasks; (**B**) differentially expressed genes; (**C**,**D**) EDGs in the foraging task and defense task gene set. mN, nectar forager of Western honeybees, *A. m. ligustica*; mP, Pollen forager; mW, water forager; mG, Guard.

The numbers of upregulated genes (log_2_FC > 1 and *p*-adjust < 0.05) and downregulated genes (log_2_FC ≤ −1 and *p*-adjust < 0.05) are presented in a bar chart (Figure 5A). Compared with pollen foragers, nectar foragers had 32 DEGs, including 15 upregulated and 17 downregulated genes. Water foragers had 74 DEGs, including 55 upregulated and 19 downregulated genes. In contrast, more DEGs were observed when compared with the defense task. The largest difference was found between pollen foragers and guard bees, with 344 DEGs (184 upregulated, 160 downregulated). A total of 8097 genes were commonly detected across all four task groups (Figure 5B). Volcano plots highlight the top 10 DEGs by adjusted *p*-value in each comparison, including genes annotated to waterproof wax synthesis (LOC411983), cGMP synthesis (LOC413596), and retinol metabolism (LOC725026) in foraging comparisons (Figure 5C), and genes annotated to olfaction (LOC408733, LOC409801) in the defense comparison (Figure 5D).

The target gene *Amfor* showed a task-dependent pattern. *Amfor* expression was significantly higher in water foragers than in both guard bees (mW vs. mG: log_2_FC = 1.2376, *p*-adj = 1.19 × 10^−18^) and nectar foragers (mN vs. mW: log_2_FC = −1.2629, *p*-adj = 0.0043), and significantly lower in nectar foragers than in guard bees (mN vs. mG: log_2_FC = −1.0768, *p*-adj = 0.0011); pollen foragers did not differ significantly from guard bees, water foragers, or nectar foragers (Table 2).

### 3.6. GO and KEGG Enrichment Analyses

**Figure 6:**
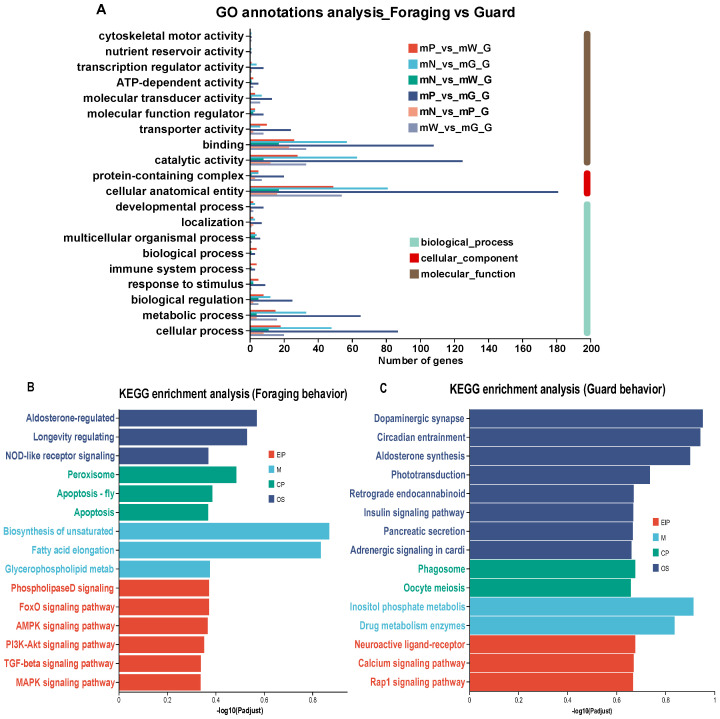
GO enrichment analysis and KEGG gene set enrichment analysis. (**A**) GO annotations analysis; KEGG enrichment analysis of foraging task (**B**) and defense task (**C**).

GO enrichment analysis was conducted on the four experimental groups to characterize significant functional DEGs related to DOL, structures, and biological pathways. The results showed that the number of significantly enriched GO terms in foraging comparisons was lower than in the guard-vs-forager comparisons (Figure 6A). Among foraging comparisons, mP vs. mW yielded the highest number of significantly enriched GO terms (129), while mN vs. mW yielded the fewest (30). Among guard-vs-forager comparisons, mP vs. mG yielded the most significantly enriched GO terms (162), and mW vs. mG the fewest (118). The top three enriched biological process (BP) terms were: (1) cellular metabolic process, (2) metabolic process, and (3) biological regulation process. The top three significantly enriched molecular function (MF) terms, ranked by adjusted *p*-value, were: (1) cytoskeletal motor activity, (2) catalytic activity, and (3) transporter activity. Under the cellular component (CC) category, GO terms related to cellular anatomical entities were enriched. In foraging behaviors, such as the mP_vs_mW gene set, enriched KEGG pathways were mainly related to environmental information processing, involving energy metabolism and cell signal transduction (Figure 6B). In defensive behaviors, organismal systems were significantly enriched in KEGG pathways (Figure 6C), mainly linked to dopamine synthesis and phototransduction.

**Figure 7:**
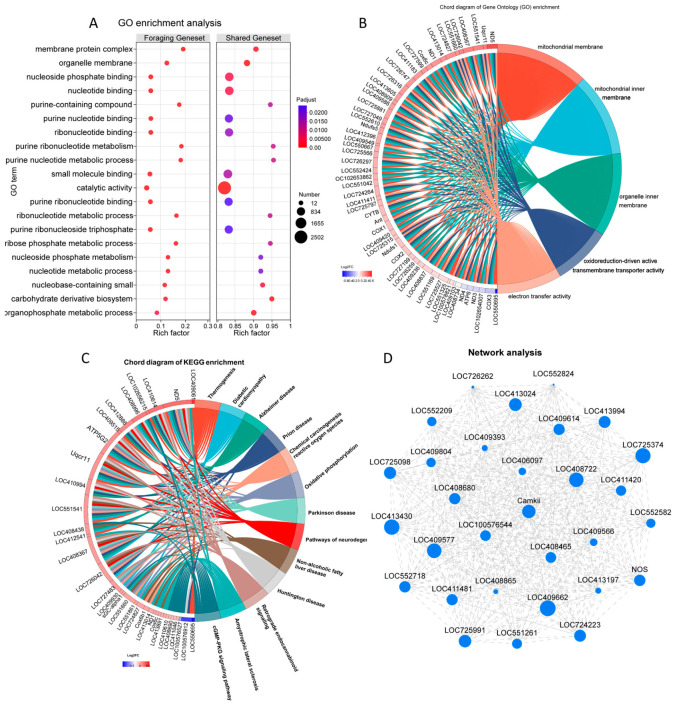
Functional enrichment and co-expression network analysis of foraging-associated DEGs. (**A**) GO enrichment analysis of DEGs from forager-subtype comparisons and commonly detected genes; (**B**) chord diagram showing the relationship between the top five enriched GO terms (*p*-adj < 0.01) and their associated DEGs; (**C**) KEGG pathway enrichment analysis of the combined foraging and guard-vs-forager DEG lists; (**D**) co-expression network of hub genes identified from WGCNA and KEGG enrichment analysis of the foraging DEG set. Note: KEGG pathways associated with neurodegenerative diseases (e.g., Alzheimer’s disease, Huntington’s disease) reflect the enrichment of mitochondrial electron transport chain and oxidative phosphorylation gene sets that are shared between these KEGG pathway maps and core metabolic pathways in *A. mellifera*, rather than disease-specific biology.

GO enrichment analysis was also performed on the combined DEG list comprising all DEGs identified in forager-subtype pairwise comparisons (mN vs. mP, mN vs. mW, mP vs. mW) across all four task groups, to characterize the shared functional landscape of foraging-associated gene expression (Figure 7A). The relationship between the top five GO entries (adjusted *p* < 0.01) and differential genes was identified (Figure 7B). Thirteen top pathways were obtained through KEGG enrichment analysis (Figure 7C). Three pathways were related to motion-regulating substances: (1) cGMP-PKG signaling pathway, (2) oxidative phosphorylation, and (3) thermogenesis. These pathways were uniquely enriched in both foraging and defensive behaviors. By comparing module identification in the foraging gene set through KEGG enrichment and Weighted Gene Co-expression Network Analysis (WGCNA, Figure 7D), it was found that *Nos* (nitric oxide synthase) and *Camkii* (calcium/calmodulin-dependent protein kinase II) were located in the same pathway, cGMP-PKG, along with genes related to cGMP-dependent protein kinase (PKG).

### 3.7. WGCNA and Gene Set Enrichment Analysis (GSEA) Analysis

**Figure 8:**
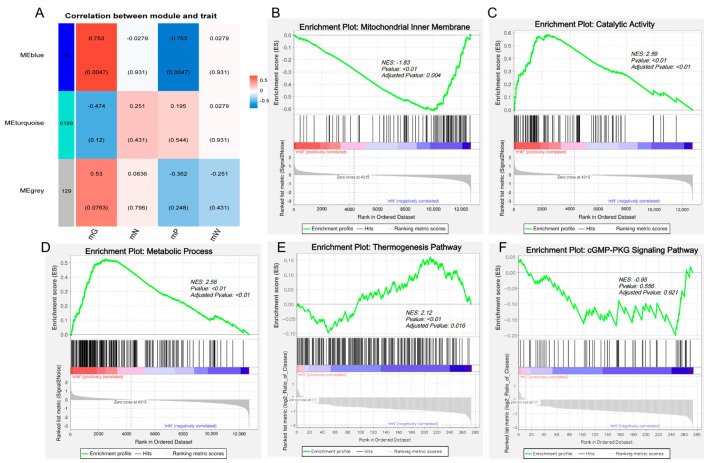
Weighted gene co-expression network analysis (**A**) and gene set enrichment analysis (**B**–**F**). mN, nectar forager; mP, Pollen forager; mW, water forager; mG, Guard.

Through WGCNA of foraging and defense tasks, three modules were identified: blue, turquoise, and grey (Figure 8A). Correlating these modules with phenotypic information on honeybee DOL revealed that the defense task was significantly positively correlated with the blue module, while the pollen-collecting task was significantly negatively correlated with the blue module. Genes with high connectivity (hub genes) within modules were subjected to correlation analysis. The results indicated that the *foraging* gene had a close regulatory relationship with both defense and foraging tasks. Through GSEA, foraging and defense tasks were associated with enrichment in the inner mitochondrial membrane (Figure 8B), catalytic activity (Figure 8C), metabolic process (Figure 8D), and thermogenesis pathway (Figure 8E). Interestingly, differences in the cGMP-PKG signaling pathway were not significant between foraging and defense tasks (Figure 8F), possibly because this pathway was required for both behaviors in the honeybee colony.

### 3.8. Validation of Key Genes by RT-PCR

**Figure 9:**
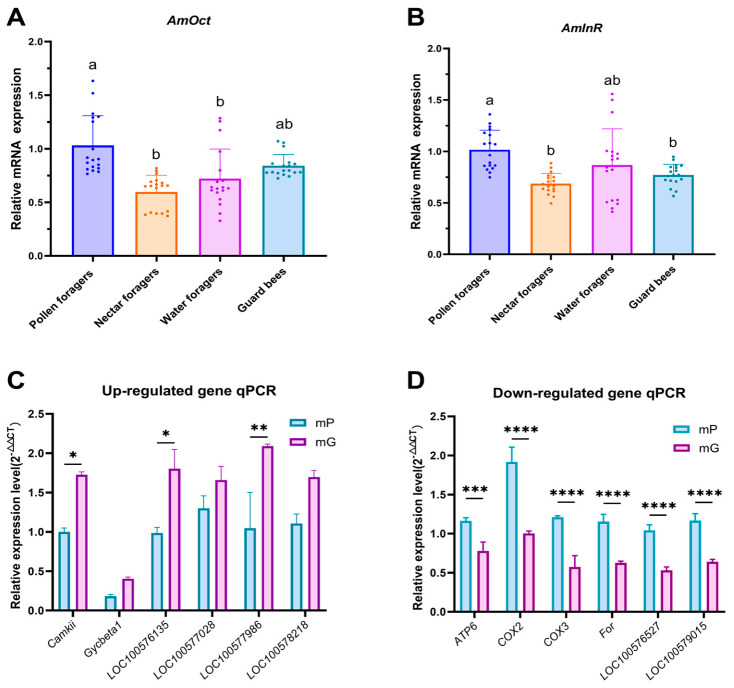
Biological and technical verification between the pollen forager group and guard group. Biological verification by qPCR of *AmOct* (**A**) and *AmInR* (**B**) gene. Technical verification by qPCR of 6 up-regulated (**C**) and down-regulated (**D**) genes. The X-axis represents the gene names, and the Y-axis represents the relative expression levels of the genes. * indicates *p* < 0.05, ** indicates 0.001 < *p* < 0.01, *** indicates *p* < 0.001, **** indicates *p* < 0.0001. Different lowercase letters (a, b) above bars indicate statistically significant differences between groups (Tukey’s HSD, *p* < 0.05); groups sharing the same letter are not significantly different.

To confirm the reliability of the RNA-seq results, we performed an independent biological validation using brain RNA extracted from six additional colonies not included in the RNA-seq analysis. Two target genes (*AmOct*, and *AmInR*) were validated by qPCR, and the results are presented in Figure 9A,B. The expression patterns of all three genes were consistent with the RNA-seq findings across task groups, confirming that the transcriptomic differences identified in the discovery phase are reproducible in an independent and larger biological sample. In addition, technical validation using the same RNA extractions as the RNA-seq libraries was performed for 12 DEGs selected from the pollen forager vs. guard bee comparison; nine of the twelve genes showed significant differential expression consistent with RNA-seq results (Figure 9C,D), and the remaining three showed concordant expression trends without reaching statistical significance. Taken together, both the independent biological validation and the technical validation support the reliability of the RNA-seq results reported in this study.

### 3.9. Hypothetical Mechanistic Framework of the cGMP-PKG Pathway in Foraging and Defense Based on Transcriptomic Evidence

**Figure 10:**
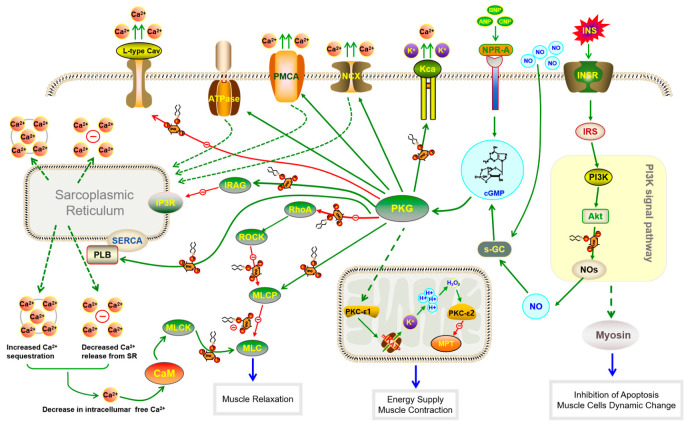
Potential mechanisms of the cGMP-PKG signaling pathway in regulating the foraging and defense of Western honeybees. The genes involved in cGMP-PKG were significantly enriched. Schematic diagram based on KEGG pathway map04022.

Based on KEGG pathway annotations and the co-expression evidence from WGCNA, in foraging behavior, natriuretic peptide family members A, B, and C act on the guanylate cyclase receptor (NPR-A) of the cell membrane (Figure 10). Upon activation, they promote intracellular cGMP production and activate PKG. Activated PKG on the cell membrane stimulates calcium-activated potassium channels (Kca) and inhibits L-type calcium channels (L-type Cav) via phosphorylation. Simultaneously, PKG acts on ATPase, PMCA, and NCX, leading to the removal of calcium and potassium ions from the cytoplasm. In the cytoplasm, activated PKG inhibits RhoA through phosphorylation. RhoA regulates Rho-associated protein kinase (ROCK), which inhibits myosin light chain phosphatase (MLCP) after phosphorylation. PKG also directly acts on MLCP, which inhibits myosin light chain (MLC) after dephosphorylation, resulting in muscle relaxation.

In the sarcoplasmic reticulum (SR), PKG phosphorylates IRAG, which inhibits IP3R, reducing calcium release from the SR. PKG also phosphorylates the SERCA-PLB complex, enhancing calcium chelation. Additionally, PKG indirectly activates PKCε1 in mitochondria, increasing mitochondrial ATP-sensitive potassium channel (mKATP) activity and altering potassium levels in the inner mitochondrial membrane. This process acts on PKCε2 and inhibits MPT, supporting energy supply for foraging and defense behaviors.

Interestingly, insulin (INS) activates the insulin receptor (INSR), which stimulates the insulin receptor substrate (IRS). Activated IRS triggers phosphatidylinositol 3-kinase (PI3K) and subsequently activates protein kinase B (Akt), initiating the PI3K pathway. Activated Akt enhances nitric oxide synthase (NOS) expression via phosphorylation, leading to nitric oxide (NO) production. Extracellular NO also enters cells and stimulates soluble guanylate cyclase (s-GC), further promoting cGMP synthesis. This pathway ultimately acts on honeybee myosin, reducing apoptosis and maintaining cellular dynamics. In defensive behavior, decreased expression of the calmodulin (CaM) gene increases calcium release from the SR and reduces calcium chelation, causing muscle contraction and prompting defensive posture in honeybees.

## 4. Discussion

This study employed behavioral, genetic, and transcriptomic approaches to investigate differences in sucrose responsiveness, gene expression, and signal pathway activity among *Apis mellifera ligustica* workers performing different task types. The results are consistent with previous studies and indicate that the *for* gene contributes to the regulation of division of labor in social insects.

### 4.1. Behavioral Differences During Labor Division

First, behavioral experiments showed that both the PER and the GRS of water foragers were significantly higher than those of nectar foragers and guard bees, indicating that these groups exhibit different response thresholds to gustatory stimuli (Figure 2). The PER directly measures reactions to tactile [38,39] and chemical stimuli [37,40], while GRS quantifies the overall intensity of gustatory responsiveness across a range of sucrose concentrations [41]. These results demonstrate that gustatory responsiveness, which includes both sensitivity and response intensity, varies systematically with task specialization in honeybees. Given the functional demands of each task: (1) water foragers require high gustatory sensitivity to detect low-concentration water signals; (2) guard bees rely more heavily on olfactory or visual cues for defense, with lower gustatory sensitivity reducing interference from non-task-related stimuli; and (3) nectar and pollen foragers need moderate gustatory responsiveness to identify chemical signals such as nectar sugar concentration and pollen volatiles. Our data suggest that variation in gustatory phenotype may function as a proximate factor that contributes to DOL in honeybee colonies. Specifically, the observed task-associated differences in PER and GRS support the hypothesis that sensory specialization aligns with task function and may guide ontogenetic or behavioral plasticity in task allocation within social colonies [42].

Social insects possess remarkable traits, including a clear DOL and highly organized social structures that are often influenced by age, genotype, and colony needs [43]. In the honeybee, *Apis mellifera*, genotype influences both the age at which a worker switches tasks and the likelihood of engaging in specialist tasks such as foraging and defense [44,45]. In this study, workers showed distinct levels of gustatory sensitivity [46] that influenced their task selection (e.g., collecting pollen, nectar, or water), thereby forming specialists in foraging or defense during behavioral development (Figure 1).

### 4.2. Differences in Relative Gene Expression Levels During Labor Division

At the molecular level, *Amfor* expression and PKG activity showed divergent patterns across task groups (Figure 3 and Table 2). PKG activity followed a clear gradient. It was highest in water foragers and lowest in guard bees. This pattern is consistent with the established role of cGMP-PKG signaling in promoting outside-hive foraging behavior [20,47]. RNA-seq analysis revealed a task-dependent pattern of *Amfor* transcript levels (Table 2). Nectar foragers showed lower *Amfor* expression than guard bees, whereas water foragers and pollen foragers showed higher expression than guard bees, with all differences below the two-fold threshold in magnitude. This suggests a dissociation between mRNA abundance and enzymatic activity. Such dissociation may reflect post-transcriptional regulation or isoform-specific differences [48,49]. Notably, both PKG activity and *Amfor* transcript levels differentiated guard bees from forager subtypes, even though both groups perform outside-hive tasks. Heylen et al. (2008) proposed that *Amfor* upregulation acts as a trigger for the nurse–forager transition [21]. Our data extend this proposal by showing that *Amfor*/PKG signaling continues to vary among outside-hive task types. This variation potentially contributes to the guard-to-forager developmental progression [23]. It is important to note, however, that *Amfor*/PKG signaling does not uniformly separate guard bees from all forager subtypes. *Amfor* may be more closely associated with the physiological and energetic demands of specific foraging modalities.

Notably, the lower expression levels of *Amfor* and PKG in guard bees (Figure 3) suggest that expression of the *foraging* gene decreases during defensive behaviour, which follows foraging behaviour in a developmental sequence. Although PKG activity has been associated with defensive behaviour in the big-headed ant (*Pheidole pallidula*) [28,50], regulatory patterns may differ among social insect species. For example, in some ant species, PKG expression patterns between nest workers and foragers show an opposite trend to that observed in honeybees [6,28,51]. In honeybees, increased *Amfor* expression and enhanced PKG activity support the transition from nurse bees to foragers [20]. The lower *Amfor* expression in guard bees relative to foragers in our data is consistent with the temporal polyethism model, in which *Amfor* upregulation marks the progression toward foraging rather than defense per se. A recent study further supports the complexity of *foraging* gene regulation across behavioral contexts in social bees, highlighting that transcript variant expression and PKG activity can be dissociated depending on task and tissue [52]. The mRNA–PKG activity dissociation we observe between guard bees (higher *Amfor* transcript in RNA-seq, lower PKG activity) warrants further investigation and may reflect post-transcriptional regulation, isoform-specific expression, or tissue-level differences in PKG activation.

### 4.3. Differentially Expressed Genes and Signaling Pathways During Labor Division

KEGG enrichment analysis identified the cGMP-PKG signaling pathway as significantly enriched in both the foraging and defensive gene sets (Figure 7C). GSEA confirmed that this pathway did not differ significantly between foraging and defense tasks (Figure 8F). These results indicate that the cGMP-PKG pathway operates as a general outside-hive behavioral enabler [53]. It is required for both resource collection and colony defense, rather than serving as a task-specific foraging switch. This is consistent with two lines of cross-species evidence. First, in *Pheidole pallidula* ants, PKG activity is elevated in defensive major workers relative to foraging minors [28]. Second, *foraging* has been shown to regulate behavioral plasticity across multiple task types via modulation of sensory thresholds [6,26]. The shared activation of this pathway across foraging and defense in honeybees may therefore reflect a common physiological requirement. Heightened sensory responsiveness, locomotor readiness, and energy mobilization are all needed for outside-hive performance, regardless of task type [54].

Defensive gene sets showed unique enrichment of dopamine synthesis and phototransduction pathways (Figure 6C). These pathways were not prominently enriched in foraging comparisons. Dopaminergic signaling is well-established as a modulator of aggression and alarm responses in insects [55]. Its specific enrichment in guard bee transcriptomes suggests that dopamine-mediated neural circuits underlie the heightened aggressive responsiveness that characterizes defensive behavior. The enrichment of phototransduction pathways in guard bees is also noteworthy. Guard bees should rapidly detect and respond to visual intrusion cues at the hive entrance [56]. Enhanced visual processing capacity may therefore represent a specific molecular adaptation for this role. Together, these pathway-level distinctions provide new molecular targets for future functional studies of guard bee specialization. They also offer a more complete picture of the transcriptomic divergence between foraging and defensive tasks.

WGCNA identified three co-expression modules associated with foraging and defensive tasks (Figure 8A). Hub gene analysis of the foraging gene set, combined with KEGG enrichment, revealed that *Nos* (nitric oxide synthase) and *Camkii* (calcium/calmodulin-dependent protein kinase II) are located in the same cGMP-PKG pathway module as *Amfor*-encoded PKG (Figure 7D). This co-regulation is mechanistically significant. NOS produces nitric oxide (NO). NO stimulates soluble guanylate cyclase (sGC) to synthesize cGMP. cGMP in turn activates PKG. This places NOS upstream of PKG within the same signaling cascade. CaMKII is activated by calcium/calmodulin and is known to interact with cGMP signaling in the regulation of synaptic plasticity and muscle function [57]. The co-expression of these three components within the same network module suggests that the functional molecular unit regulating task-specific behavior is not *Amfor* alone. It is a broader calcium-NO-cGMP signaling network. This network-level view is more precise than the single-gene framing of prior candidate gene studies [20,21,47]. It identifies *Nos* and *Camkii* as priority targets for future pharmacological or genetic manipulation in the context of honeybee DOL.

GSEA consistently identified enrichment of inner mitochondrial membrane components (Figure 8B), metabolic processes (Figure 8D), and thermogenesis pathways (Figure 8E) across both foraging and defensive tasks. This pattern suggests that outside-hive activity imposes a shared metabolic signature. This signature is distinct from in-hive tasks and is independent of whether the outside-hive role is foraging or guarding [58]. The thermogenesis enrichment is particularly relevant to honeybee thermoregulation. Foragers and guard bees both operate at the hive entrance or in the field. Ambient temperature fluctuations are greater there than inside the hive, and sustained flight muscle activity is required [59]. The mitochondrial and metabolic enrichment we observe may therefore reflect an adaptive upregulation of energy production capacity common to all outside-hive workers.

Several limitations of this study should be acknowledged. First, the use of three colonies as biological replicates, while consistent with standard practice in honeybee transcriptomics, limits the generalizability of findings across broader genetic backgrounds and colony conditions. Colony-level variation in *Amfor* expression and PKG activity may exist and warrants investigation in larger colony samples. Second, bees were assigned to task groups based on behavioral observation rather than chronological age tracking. In *A. mellifera*, behavioral state and chronological age are strongly correlated under natural conditions, and we cannot fully exclude the possibility that some transcriptomic differences between guard bees and foragers reflect age-related gene expression changes rather than task-specific regulation alone. Future studies using paint-marked newly emerged bees tracked longitudinally would allow behavioral and chronological age effects to be formally separated. Third, the experience level of captured foragers was not controlled. Nectar foragers were collected after stable feeder recruitment was established, predominantly capturing experienced recruits; however, scout bees cannot be entirely excluded. For water foragers, prior foraging history (e.g., previous nectar or pollen collection) was not verified. This introduces potential noise into behavioral classification and may contribute to within-group transcriptomic variance. Fourth, the proposed calcium-NO-cGMP network model is based on co-expression and pathway enrichment data; causal relationships among *Nos*, *Camkii*, and *Amfor*/PKG remain to be established through functional genetic studies.

Taken together, these findings support a revised model of DOL regulation. In this model, *Amfor*-mediated cGMP-PKG signaling operates as one node within a broader calcium-NO-cGMP network. Task-specific differentiation is achieved through additional pathway-level differences, particularly dopaminergic and phototransduction circuits in guard bees. Mitochondrial metabolic reprogramming constitutes a shared physiological foundation for outside-hive behavioral performance. This model is more precise than the single-gene framing and provides a clearer roadmap for future mechanistic studies of social insect DOL.

## 5. Conclusions

This study reveals that *Amfor*-mediated cGMP-PKG signaling functions as a shared outside-hive behavioral enabler for both foraging and defense in *Apis mellifera ligustica*, rather than a foraging-specific switch. Dopamine synthesis and phototransduction pathways specifically distinguish guard bee transcriptomic signatures, while *Nos* and *Camkii* co-regulate with PKG within a broader calcium-NO-cGMP network. Mitochondrial metabolic reprogramming constitutes a shared physiological foundation for all outside-hive tasks. These findings provide a transcriptomic framework for understanding the molecular regulation of division of labor in social insects.

## Figures and Tables

**Table 1:** Quality metrics of transcripts in the labor task of honeybee, *A. m. ligustica*.

Sample	Raw Reads	Clean Reads	GC Content (%)	Total Reads	Uniquely Mapped
mN_1	47,847,188	47,823,746	38.71	47,823,746	44,898,397 (93.88%)
mN_2	53,619,536	53,593,208	37.82	53,593,208	50,317,735 (93.89%)
mN_3	56,506,700	56,477,856	37.64	56,477,856	52,313,549 (92.63%)
mP_1	57,573,986	57,546,724	38.49	57,546,724	32,877,123 (87.13%)
mP_2	52,115,004	52,088,858	37.82	52,088,858	48,792,829 (93.67%)
mP_3	59,167,604	59,138,074	38.39	59,138,074	50,865,069 (86.01%)
mW_1	51,916,632	51,890,840	38.37	51,890,840	47,912,738 (92.33%)
mW_2	45,349,602	45,327,254	39.14	45,327,254	42,427,299 (93.60%)
mW_3	59,369,126	59,340,992	36.92	59,340,992	55,696,234 (93.86%)
mG_1	56,188,244	56,162,196	38.43	56,162,196	52,341,396 (93.20%)
mG_2	57,465,022	57,437,224	39.05	57,437,224	52,246,445 (90.96%)
mG_3	57,866,000	57,837,916	38.33	57,837,916	53,334,791 (92.21%)

mN, nectar forager of Western honeybees, *A. m. ligustica*; mP, Pollen forager; mW, water forager; mG, Guard. GC is the percentage of G and C bases in all transcripts.

**Table 2:** Log2 fold change in *foraging* genes encoded cGMP-dependent protein kinase *foraging*, transcript variant X1 in the labor task.

Labor Task	Log_2_FC	*p* Adjust	Significant	Regulate
mN_vs_mW	−1.2629	0.0043	yes	down
mN_vs_mP	0.1348	0.8807	no	up
mW_vs_mG	1.2376	1.1872 × 10^−18^	yes	up
mP_vs_mW	−0.2039	2.0588 × 10^−16^	no	down
mP_vs_mG	0.0955	0.0010	no	up
mN_vs_mG	−1.0768	0.0011	yes	down

Although the adjusted *p*-values for several pairwise comparisons of *Amfor* were below 0.05, none of these comparisons reached statistical significance under the threshold of lfcThreshold = 1. This is because the observed |log_2_FC| values fell within the null hypothesis range of |LFC| ≤ 1.

## Data Availability

The raw data obtained from these observations will be uploaded and made available at a Mendeley Data repository (doi: 10.17632/zktrrn69xt.2).

## Supplementary Materials

The following supporting information can be downloaded at: https://www.mdpi.com/article/10.3390/insects17060583/s1, Table S1: Primer oligonucleotides based on mRNA sequences.
